# Quantum transport of massless Dirac fermions through wormhole-shaped curved graphene in presence of constant axial magnetic flux

**DOI:** 10.1038/s41598-024-57718-3

**Published:** 2024-04-02

**Authors:** F. Naderi, K. Hasanirokh

**Affiliations:** 1 Young Researchers and Elite Club, Marand Branch, Islamic Azad University, Marand, Iran; 2 Department of Physics, Faculty of Basic Sciences, Azarbaijan Shahid Madani University, Tabriz, 53714-161 Iran

**Keywords:** Condensed-matter physics, Electronics, photonics and device physics, Quantum physics

## Abstract

In this work, we have studied the spin-dependent quantum transport of charged fermion on $(2+1)$-dimensional spacetime, whose spatial part is described by a wormhole-type geometry in the presence of constant axial magnetic flux. Choosing the solutions of the Dirac equation associated with real energy and momentum, we explored the spin-dependent transmission probabilities and giant magnetoresistance (GMR) through a single layer of wormhole graphene with an external magnetic field, using the transition matrix (*T*-Matrix) approach. The spin-up and spin-down components within the *A* and *B* sublattices of graphene in the matrix of $4\times 1$ wave function are coupled to each other due to the wormhole structure and the magnetic field. We have found that transport properties strongly depend on the magnetic field, incident energy, and geometric parameters of the system. We observed that the transmission probability increases as the radius of the wormhole increases, and the length of the wormhole decreases. The higher energies lead to a decrease in the transmission probabilities of particles. Furthermore, we observed that the probability of the spin-flip effect is almost larger than that of the non-spin-flip effect, illustrating that electrons lose their spins during transmission. These findings highlight the complex and interesting behavior of wormhole graphene in the presence of external magnetic fields and suggest that these nano structures can have potential applications in electronic and spintronic devices.

## Introduction

In recent years, the study of fermions on 2-dimensional surfaces has been a significant area of research. This field has revealed intriguing boundary phenomena that do not have analogs in the bulk, such as the quantum (spin) Hall effect, topological matters, and the physics of graphene^1^. The physics of graphene has led to numerous practical applications in various fields. Graphene’s high electrical conductivity and strength make it an ideal material for the development of faster and more efficient electronic devices. Graphene-based transistors, for example, have the potential to revolutionize the field of electronics by enabling faster and more energy-efficient computers. In addition, graphene’s unique properties also make it a promising material for energy storage and conversion. Graphene-based batteries and supercapacitors have shown promise for high-capacity energy storage, while graphene-based solar cells have the potential to be more efficient and cheaper than traditional silicon-based solar cells^2–4^. Graphene sheets are highly flexible and can be curved, rolled, stretched, twisted, and deformed without losing their unique properties. Even puncturing holes into graphene sheets is possible, and these holes can be connected to nanotubes to create a curved structure called wormhole that bridges two graphene sheets. By connecting multiple wormholes, a network of entangled electronic structures can be formed, allowing multiple graphene sheets to be interconnected with each other^5^. Researchers have even produced cage structures of graphene wormholes called “schwarzites,” which have promising properties^6,7^.

Generally, the studies on curved graphene take advantages of the gauge field theory approaches, where interpreting the geometrical curvature of the 2D structure as pseudo gauge fields, the Dirac field equation in a $(2+1)$-dimensional curved spacetime provides the dynamics of quasi-particles dynamics in graphene^8–13^. In fact, presence of gauge fields can significantly impact the behavior of quantum particles on a curved surface^8,13^. By applying gauge fields, such as electric and magnetic fields, to these systems, unique and intriguing 2*D* phenomena can emerge, including the well-known Landau quantization of fermionic states on a plane^14,15^. It has been demonstrated that the effects of strain and gauge fields can be similar in 2D^8,16^. The geometric curvature effects of 2D surface can mimic gauge fields, and the curvature and gauge connection appear with equal importance in the equation of motion^17–21^. The behavior of quantum particles on a curved surface in the presence of gauge fields can be significantly different from the flat situation^22,23^. For instance, fermions on a 2-dimensional sphere and wormhole experience spin-orbit coupling induced by the surface curvature, even in the absence of extra gauge fields^24^. Adding an axial magnetic field, generates Landau quantization that is distinct from the planar case^25^. Particularly, the graphene wormholes in presence of an external magnetic field have been studied in^25–28^.

Beside graphene, wormhole surface has been also considered in further 2D structures, for instance the liquid crystal film which has been studied to construct optical wormhole from hollow disclinations in^29^. Meanwhile, electromagnetic wormholes and virtual magnetic monopoles have also attracted the attention of many researches. Based on the Maxwell’s equations, the tunneling of electromagnetic wave between two points has been investigated in these devices. The growing development of metamaterials designed for microwave and optical applications such as optical cables, 3D video displays and optical computers requires the study of the electromagnetic wormholes^30^. It should be noted that the 2-dimensional wormhole is fundamentally different from those appear in $(3+1)$-dimensional spacetimes in General Relativity (see, for example,^31^), as there is no time dilation in the 2-dimensional wormhole under consideration.

The unique properties of quantum particles on curved surfaces in the presence of gauge fields have led to the development of several promising technologies, such as topological quantum computing, quantum sensors, and quantum communication. In this area, the study of charged fermions in wormholes is a relatively new area of research, and practical applications are still being explored. However, there are some potential applications that could emerge from this research in the future. One possible application is in the development of new materials with unique electronic properties. The behavior of charged fermions in a wormhole is influenced by the geometry of the wormhole, which can be controlled in certain materials. This could potentially lead to the development of materials with novel electronic properties that could be useful in a wide range of applications, from electronics to energy storage. Another possible application is in the development of new quantum technologies. The behavior of charged fermions in a wormhole can exhibit quantum effects such as entanglement, which could potentially be useful for the development of new quantum technologies such as quantum computing and quantum communication. There are several challenges that researchers face when studying charged fermions in wormholes: mathematical complexity, experimental limitations, unconventional behavior, and limited understanding of wormholes.

The study of GMR has numerous practical applications in the field of spintronics. GMR refers to the phenomenon where the electrical resistance of a magnetic material changes significantly in response to an external magnetic field. This effect can be exploited in the design of magnetic sensors, magnetic random access memory (MRAM), and other spintronics devices. One practical application of GMR is in the development of hard disk drives (HDDs). GMR sensors are used to read the magnetic data stored on the disk platters in an HDD. The GMR effect allows for higher data storage densities and faster data transfer rates, making HDDs more efficient and cost-effective.

The behavior of Dirac fermions in graphene wormholes without gauge fields has been discussed in Ref.^5,32^. In Ref.^25,26^, authors investigated the effects of an axial magnetic field on a charged fermion in a $(2 + 1)$-dimensional wormhole spacetime. For the scenario of a constant magnetic flux, the system can be solved analytically to provide exact solutions that contain both “normal” modes (real energy but complex momentum) and quasinormal modes.

Our research focuses on exploring the spin-dependent quantum transport characteristics of a charged fermion as it travels through the surface of a wormhole, while an external magnetic field is present along the axis direction of the wormhole. Following the formalism presented in^26^, we first extend the class of solutions for Dirac equation to provide the wave functions to be used in *T*-matrix method. Then, limiting our attention to the solutions with real energy and momentum, the spin-dependent transmission probabilities and giant magnetoresistance (GMR) through a single layer of wormhole graphene in the presence of an external magnetic field are studied.

In “Theoretical model and calculations” section, the mathematical formalism is established to obtain the solutions of Dirac equation on the $(2+1)$-spacetime constructed by the wormhole type spatial part, tacking into the account the magnetic field associated with a constant axial flux. Then, “Results and discussion” section contains the discussion of the obtained results. Finally, some concluding remarks are presented in “Conclusion” section.

## Theoretical model and calculations

The curved graphene structures can be considered as 2D pseudo-relativistic systems, which in the long wavelength continuum limit at which graphene sheet can be seen as a continuum with characteristic Fermi velocity $v_F$, is described by massless Dirac action, whose variation gives the equations of motion for the pseudo-relativistic Dirac spinors $\Psi$^8–11,33^. The curvature of spacetime is usually interpreted as an effective potential experienced by the fermion, which appears in the Dirac equation by modifying the partial derivative.

In this section, providing the formalisim of massless Dirac equation on $(2+1)$-dimensional curved wormhole spacetime in presence of a magnetic field with constant axial flux, the four dimensional Dirac spinor are obtained by solving the massles Dirac equation. Also, matching conditions in the scattering are studied in the *T*-Matrix approach.

### Dirac equation on curved spacetimes

The general form of Dirac equation on curved spacetime in the presence of an additional electromagnetic four-potential $A_{\mu }$ is^33^

1 $$\begin{aligned} \hat{\gamma }^{a}e^{\mu }_{a}\left( -\hbar \nabla _{\mu }+i\frac{e}{c}A_{\mu }\right) \Psi = 0, \end{aligned}$$

where $\Psi$ represents the Dirac spinor field, *e* is electric charge, $a=0,1,2$ are flat coordinate basis indices, $\mu =0,1,2$ are curved spacetime indices, $\hat{\gamma }^{a}$ denote flat $\gamma$-matrices, ${\nabla }_\mu$ is the covariant derivative, and $e_{\mu }^a$ are the vielbeins, which are obtained by writing the curved spacetime metric $g_{\mu \nu }$ in terms of flat Minkowski metric, $\eta _{ab}=\textrm{diag}(-1,1,1)$ in the following form

2 $$\begin{aligned} g_{\mu \nu }(x) = e_\mu ^{~a}(x)\;e_\nu ^{~b}(x)\;\eta _{ab}. \end{aligned}$$

Defining ${\hat{\gamma }}^a=\eta ^{ab}\,{\hat{\gamma }}_b$, in which $\eta ^{ab}$ denotes the inverse of the Minkowski metric, the ${\hat{\gamma }}^a$ matrices should satisfy the standard Clifford algebra $\left\{ {\hat{\gamma }}^a,{\hat{\gamma }}^b\right\} =2\,\eta ^{ab}\,\mathbb {1}$. The covariant derivative ${\nabla }_\mu$ is defined as follows^33^

3 $$\begin{aligned} \nabla _{\mu }\equiv \partial _{\mu }-\Gamma _{\mu }, \end{aligned}$$

where $\Gamma _\mu$, standing for the spin connection of the spinor field, plays the role of an effective gauge field and is given by

4 $$\begin{aligned} \Gamma _{\mu }=-\frac{1}{4} \hat{\gamma }^{a}\hat{\gamma }^{b} e^{\nu }_{a} \left[ \partial _{\mu }\left( g_{\nu \beta }e^{\beta }_{b}\right) -e^{\beta }_{b}\Gamma _{\beta \mu \nu } \right] , \end{aligned}$$

where the Christoffel symbols $\Gamma _{\beta \mu \nu }$ are defined by

$$\begin{aligned} \Gamma _{\beta \mu \nu }=\frac{1}{2}\left( \partial _{\mu }g_{\beta \nu }+\partial _{\nu }g_{\beta \mu }-\partial _{\beta }g_{\mu \nu }\right) . \end{aligned}$$

Considering the $(2+1)$-dimensional curved spacetime to be described by the line element

5 $$\begin{aligned} ds^{2}= g_{\mu \upsilon } dx^{\mu } dx^{\upsilon }=-c^{2}dt^{2}+du^{2}+R^{2}(u)d v^{2}, \end{aligned}$$

the non-zero components of $\Gamma _{\beta \mu \nu }$ are

6 $$\begin{aligned} -\Gamma _{uvv} = \Gamma _{vuv} = \Gamma _{vvu}=\frac{1}{2}\partial _{u}R^{2}=RR', \end{aligned}$$

which lead to

7 $$\begin{aligned} \Gamma _{t}=0, \quad \Gamma _{u}=0, \quad \Gamma _{v}=\frac{1}{2}\hat{\gamma }^{1}\hat{\gamma }^{2}R'. \end{aligned}$$

For the Dirac matrices, the following representation will be adopted

$$\begin{aligned} \hat{\gamma }^{0}=\left( \begin{array}{cc} i &{} 0 \\ 0 &{} -i \\ \end{array} \right) ,\quad \hat{\gamma }^{k}=\left( \begin{array}{cc} 0 &{} i\sigma ^{k} \\ -i\sigma ^{k} &{} 0 \\ \end{array} \right) , \end{aligned}$$

where $\sigma ^{k}$ are the Pauli matrices. The $\gamma$ matrices obey the Clifford algebra $\{\hat{\gamma }^{a}, \hat{\gamma }^{b}\}=2\eta ^{ab}$, using the identity of Pauli matrices $\sigma ^{i}\sigma ^{j}=\delta ^{ij}+i\epsilon ^{ijk}\sigma ^{k}$, in which $\epsilon ^{ijk}$ is Levi-Civita symbol.

### Geometric and gauge description of the wormhole

**Figure 1:**
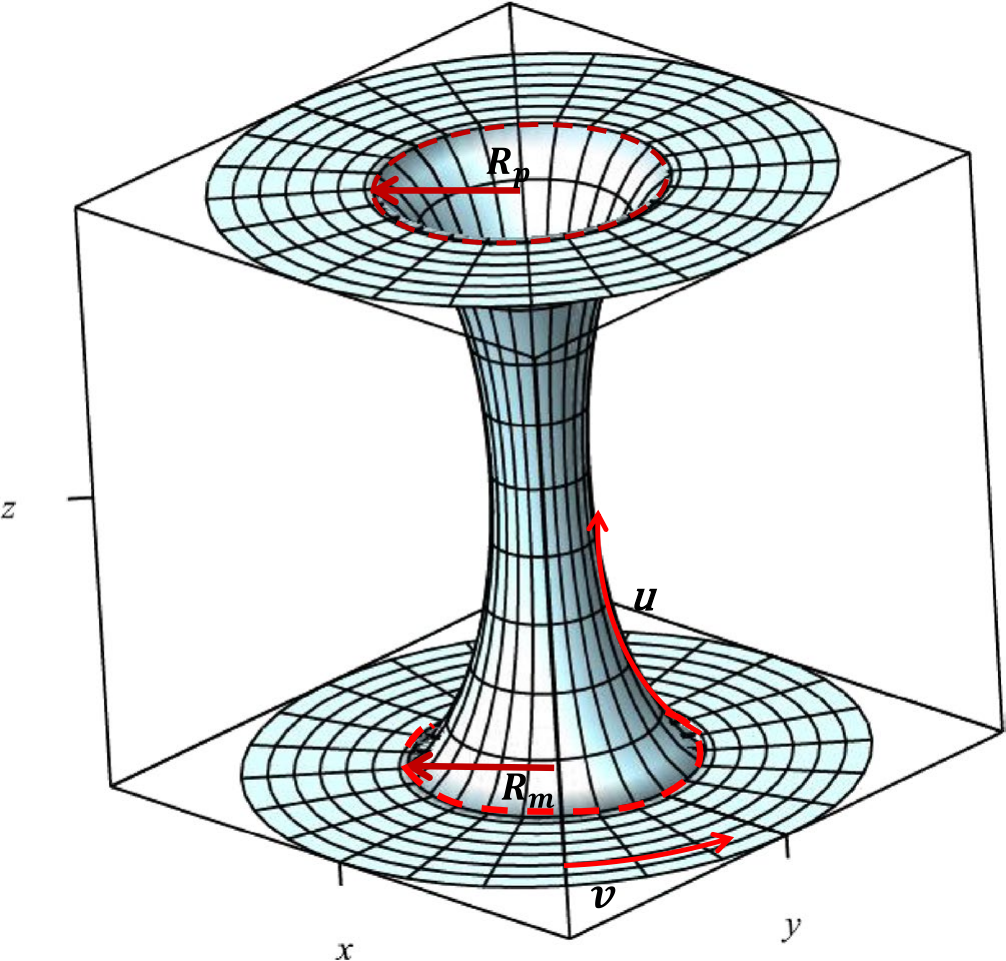
A wormhole created by smooth connection of a hyperbolic bridge to two flat planes at $u=u_{p}$ and $u=u_{m}$.

We follow the geometric and gauge setup provided in^26^, where the curved graphene is considered to be constructed by two flat planes connected by a hyperbolic bridge, as shown in Fig. 1. In doing so, the metric (5) is described by the following *R*(*u*) function

8 $$\begin{aligned} R(u)=\Bigg \{\begin{array}{cc}\begin{aligned} u-u_{p}+R_{p},\quad \quad \quad \quad \textrm{for}\quad u_{p}<u_p \\ a\cosh _{q}(u/r)\quad \quad \textrm{for}\quad u_p\le u \le u_{m}\\ -(u-u_{m})+R_{m}, \quad \quad \textrm{for}\quad u<u_{m},\\ \end{aligned}\end{array} \end{aligned}$$

in which *a* and *q* are constants, $\cosh _q(x)\equiv \frac{e^{x}+qe^{-x}}{2}$ is the *q*-deformed hyperbolic function defined by^34^, and $u_{p,m}$ denote the values of *u* at which the wormhole ends and connects to the upper and lower planes, where $R'(u_{p,m})=\pm 1$, and

9 $$\begin{aligned} u_{p,m}=r\ln \displaystyle {\Big (\sqrt{q+\frac{r^2}{a^2}}\pm \frac{r}{a}\Big )} \quad \quad \textrm{and}\quad \quad R_{p,m}=\sqrt{qa^{2}+r^{2}}. \end{aligned}$$

$R_{p,m}\equiv R(u_{p,m})$ stand for the corresponding radial distance at the boundary of the upper and lower plane. At the midpoint of wormhole located at $u_{0}=\frac{1}{2}\ln q$, $R(u)=a$ that is the minimum of *R*(*u*) function. The wormhole is symmetric with respect to $u_{0}$. Also, the *v* coordinated is in the range of $v\in [0,2\pi ]$. When $q=1$, the deformed hyperbolic functions reduce to hyperbolic functions.

To apply an external magnetic field with constant flux through the circular area enclosed by the wormhole at a fixed *z*, the four-potential in the wormhole coordinates is considered to be^26^

10 $$\begin{aligned} A_{\mu }(t,u,v)=\frac{\partial _{}x^{\nu '}}{\partial _{}x^{\mu }}A_{\nu '}(t,x,y,z)=\left( 0,0,\frac{1}{2}BR^{2}\right) , \end{aligned}$$

whose associated magnetic filed in *z* direction is $B_z\propto \frac{1}{R^2}$, leads to constant magnetic flux, $\phi =\int \vec {B}.d\vec {a}=\pi R^2 B$.

### Dirac equation in the wormhole in presence of constant magnetic flux

In the described geometry, the Dirac equation (1) reads^26^

11 $$\begin{aligned} \left( \begin{array}{cc} i\partial _{ct} &{} i{\textbf {D}} \\ -i{\textbf {D}} &{} -i\partial _{ct} \\ \end{array}\right) \Psi =0, \end{aligned}$$

where ${\textbf {D}}$ is a differential operator

12 $$\begin{aligned} {\textbf {D}}\equiv \sigma ^{1}\left( \partial _{u}+ \frac{R'}{2R}\right) +\sigma ^{2}\left( \frac{1}{R}\partial _{v}-\frac{ie}{2\hbar c}BR\right) . \end{aligned}$$

Considering a stationary state of the Dirac spinor

13 $$\begin{aligned} \Psi (t,u,v)=\textrm{e}^{-\frac{i}{\hbar }Et}\textrm{e}^{imv}\left( \begin{array}{cc} \chi _{up}(u) \\ \chi _{d}(u) \\ \varphi _{up}(u)\\ \varphi _{d}(u) \end{array} \right) , \end{aligned}$$

where $m=0,\pm 1,\pm 2, \ldots ,$ is the orbital angular momentum quantum number, and the *up* and *d* indices refer to spins up and down, respectively, the equation (11) leads to the following coupled set of equations

14 $$\begin{aligned}{} & {} {\varphi _{up}'}(u)+{\frac{ { R'}-2\,{m'} }{ R}}{ \varphi _{up}}(u)+\frac{iE}{\hbar c}\chi _d(u)=0, \end{aligned}$$

15 $$\begin{aligned}{} & {} {\varphi _d'}(u)+{\frac{ { R'}+2\,{m'} }{ R}}{ \varphi _d}(u)+\frac{iE}{\hbar c}\chi _{up}(u)=0, \end{aligned}$$

16 $$\begin{aligned}{} & {} {\chi _{up}'}(u)+{\frac{ { R'}-2\,{m'} }{ R}}{ X_{up}}(u)+\frac{iE}{\hbar c}\varphi _d(u)=0, \end{aligned}$$

17 $$\begin{aligned}{} & {} {\chi _d'}(u)+{\frac{ { R'}+2\,{m'} }{ R}}{ X_d}(u)+\frac{iE}{\hbar c}\varphi _{up}(u)=0, \end{aligned}$$

where $m'=m-\displaystyle {\frac{\phi }{\phi _{0}}}$, $\phi$ is the constant magnetic flux, and $\phi _{0}\equiv hc/e$ is the magnetic flux quantum. Combining these equations, one can get the following equation for $\varphi (u)$ functions

18 $$\begin{aligned} \begin{aligned} \varphi ''(u) +&\frac{R'}{R}\varphi '(u) +\bigg (\frac{R''}{2R}-\left( \frac{R'}{2R}\right) ^2 +\frac{m'\sigma R'-m'^2}{R^2}+k^2\bigg ) \varphi (u)=0, \end{aligned} \end{aligned}$$

where $\sigma$ is the spin-state index, and $\varphi _{up}(u)$ and $\varphi _d(u)$ are associated with $\sigma =+1$ and $\sigma =-1$, respectively. Also, $k^{2} \equiv E^2/\hbar ^{2}c^{2}$ is the momentum parameter.

The $\phi _{up}(u)$ and $\phi _{d}(u)$ functions obtained by solving the equation (18) can be used to determine the the $\chi (u)$ functions, via equations (16) and (17).

### Solutions in the hyperbolic throat of wormhole

The equation (18) have been solved in^26^ by defining the variable $X(u)\equiv rR'(u)/a=\sinh _{q}(u/r)$ and considering weighting function solution $\varphi (X)=(\sqrt{q}+iX)^\alpha (\sqrt{q}-iX)^\beta \Phi (X)$. Setting $X=-i\sqrt{q}Y$, the equation (18) is then rewritten in terms of *Y* as follows

19 $$\begin{aligned} \begin{aligned}{}&(1-Y^2)\Phi ''(Y)+2\Big [(\alpha -\beta )-(\alpha +\beta +1)Y\Big ]\Phi '(Y)-\Big [(\alpha +\beta )(\alpha +\beta +1)+k^2 r^2+\frac{1}{4}\Big ]\Phi (Y)=0, \end{aligned} \end{aligned}$$

which is the Jacobi Differential Equation. The energy levels are^26^

20 $$\begin{aligned} E^{2}_{n,m'}=\hbar ^{2}c^{2}k^{2}_{n,m'} =-\frac{\hbar ^{2}c^{2}}{r^2}\left( n+\frac{1}{2}+\alpha +\beta \right) ^{2}, \end{aligned}$$

where *n* is a real constant and the powers in the weight factors of $\phi$ functions are

21 $$\begin{aligned} \alpha =\kappa _{1}\left( \frac{1}{4}+\frac{i}{\sqrt{q}}\frac{\sigma m'r}{2a}\right) , \,\quad \beta =\kappa _{2}\left( \frac{1}{4}-\frac{i}{\sqrt{q}}\frac{\sigma m'r}{2a}\right) , \end{aligned}$$

in which $\kappa _{1},\kappa _{2}=\pm 1$. We consider the solution of (18) given by the following combination(The term associated with $C_2$ is not included in the solutions provided in^25,26^)

22 $$\begin{aligned} \begin{aligned} \varphi _{n,m',\sigma }(\kappa _{1},\kappa _{2},u)=&\left( \sqrt{q}+iX\right) ^{\alpha } \left( \sqrt{q}-iX\right) ^{\beta }\times \bigg (A_{\kappa _{1},\kappa _{2},\sigma }P_{n}^{(2\alpha ,2\beta )} \left( \frac{iX}{\sqrt{q}}\right) \\&+B_{\kappa _{1},\kappa _{2},\sigma } \left( {IX}{}+\sqrt{q}\right) ^{2\alpha }P_{-n-1-2\beta }^{(-2\alpha ,2\beta )}\left( \frac{iX}{\sqrt{q}}\right) \bigg ), \end{aligned} \end{aligned}$$

where *A* and *B* are constants. On the other hand, by rewriting the equations (16) and (17) in terms of *X* as follows

23 $$\begin{aligned} \begin{aligned} \chi _{up}(X)&={\frac{i }{kra\sqrt{{X}^{2}+1}}}\bigg ( a \left( {X}^{2}+q \right) \varphi _d' \left( X \right) +\frac{1}{2} \left( 2\,{m'}\,r+X a \right) \bigg )\varphi _d \left( X \right) , \end{aligned} \end{aligned}$$

24 $$\begin{aligned} \begin{aligned} \chi _d(X)&={\frac{i }{kra\sqrt{{X}^{2}+1}}}\bigg ( a \left( {X}^{2}+q \right) \varphi _{up}' \left( X \right) +\frac{1}{2} \left( - 2\,{m'}\,r+X a \right) \bigg )\varphi _{up} \left( X \right) , \end{aligned} \end{aligned}$$

we obtain the $\chi _{up}(u)$ and $\chi _{d}(u)$ functions by substituting (22) into (23) and (24) as follows

25 $$\begin{aligned} \begin{aligned}{}&\chi _{n,m',\sigma }(\kappa _{1},\kappa _{2},u)={\frac{i \left( \sqrt{q}-iX \right) ^{\beta } \left( iX+\sqrt{q} \right) ^{-\alpha }}{ \left( {X}^{2}+q \right) ^{\frac{3}{2}}rak}} \bigg [ A_{\kappa _{1},\kappa _{2},\sigma }{P_n } ^{(2 \alpha ,2 \beta )}\left( {\frac{-iX}{\sqrt{q}}} \right) \bigg ( ia \left( \alpha +n+3 \beta +1 \right) {q}^{\frac{3}{2}}\\&\quad \quad +ia \left( \alpha +n+3 \beta +1 \right) {X}^{2}\sqrt{q}+ \left( X \left( \frac{1}{2}+\alpha +\beta +n \right) a-{m'} r\sigma \right) \left( {X}^{2}+q \right) \bigg ) \\&\quad \quad -A_{\kappa _{1},\kappa _{2},\sigma }{ P_n}^{(2 \alpha ,2 \beta +1)} \left( {\frac{-iX}{\sqrt{q}}} \right) a \left( i{q}^{\frac{3}{2}}+ \left( i\sqrt{q}X+{X}^{2}+q \right) X \right) \left( 2 \alpha +n+2 \beta +1 \right) \\&\quad \quad -2B_{\kappa _{1},\kappa _{2},\sigma }{P}_{-2 \beta -n-1}^{(-2 \alpha ,2 \beta +1)} \left( {\frac{-iX}{\sqrt{q}}} \right) a \left( i{q}^{\frac{3}{2}}+ \left( i\sqrt{q}X+{X}^{2}+q \right) X \right) \left( \alpha +\frac{n}{2} \right) \\&\quad \quad +B_{\kappa _{1},\kappa _{2},\sigma }{P}_{-2 \beta -n-1}^{(-2 \alpha ,2 \beta )} \left( {\frac{-iX}{ \sqrt{q}}} \right) \bigg ( ia \left( \alpha +n-\beta \right) {q}^{\frac{3}{2}} +ia{X}^{2} \left( \alpha +n-\beta \right) \sqrt{q}\\&\quad \quad \quad \quad + \left( X \left( \frac{1}{2}+\alpha +\beta +n \right) a+r\sigma { m'}\right) \left( {X}^{2}+q \right) \bigg ) \bigg ], \end{aligned} \end{aligned}$$

where $\sigma =\pm 1$ are associated with $\chi _{up}$ and $\chi _d$, respectively.

Depending on the sign choices of $\kappa _{1}$ and $\kappa _{2}$, the the spin-orbit coupling term $\sim \sigma mr/a\sqrt{q}$ can be present or absent at the energy levels described by (20), in such a way that^26^

26 $$\begin{aligned} k_{n,m'}r=i\left( n+1\right) ,\quad \quad \quad \quad E^2=-\frac{\hbar ^{2}c^{2}}{r^2}(n+1)^2, \end{aligned}$$

for $$\kappa _{1}=\kappa _{2}=1$$,

27 $$\begin{aligned} k_{n,m'}r=in,\quad \quad \quad \quad E^2=-\frac{\hbar ^{2}c^{2}}{r^2}n^2, \end{aligned}$$

for $$\kappa _{1}=\kappa _{2}=-1$$,

28 $$\begin{aligned} \begin{aligned} k_{n,m'}r&=i\left( n+\frac{1}{2}\right) -\frac{\sigma m'r}{a\sqrt{q}},\quad \quad \quad \quad E^2=-\frac{\hbar ^{2}c^{2}}{r^2}\left( n+\frac{1}{2}+i\frac{\sigma m'r}{a\sqrt{q}}\right) ^{2}, \end{aligned} \end{aligned}$$

for $$\kappa _{1}=-\kappa _{2}=1$$, and

29 $$\begin{aligned} \begin{aligned} k_{n,m'}r&=i\left( n+\frac{1}{2}\right) +\frac{\sigma m'r}{a\sqrt{q}},\quad \quad \quad \quad E^2=-\frac{\hbar ^{2}c^{2}}{r^2}\left( n+\frac{1}{2}-i\frac{\sigma m'r}{a\sqrt{q}}\right) ^{2}, \end{aligned} \end{aligned}$$

for $\kappa _{1}=-\kappa _{2}=-1$. Accordingly, the momentum *k* is generally complex-valued, which shows that the solutions are quasi-normal modes. As it is shown in^26^, the solutions can be analytically continued to the cases with negative *n*, where the $n=-\frac{1}{2}$ yields normal modes with real energy and momentum in the (28) and (29) cases.

Hence, in the hyperbolic bridge with $u_{m}\le u\le u_{p}$, where the components of $\Psi$ are given by (22) and (25), the general solution with real energy can be expressed as

30 $$\begin{aligned} \begin{aligned} \Psi (u)= \Psi _{m,n,\sigma }(+,-,u)+\Psi _{m,n',\sigma '}(-,+,u), \end{aligned} \end{aligned}$$

where $X=\sinh _{q}(u/r)$, the ± signs denote the values of $\kappa _1$ and $\kappa _{2}$ chosen to have real values *k* and *E*, and the condition $E_{n,\sigma }=E_{n',\sigma '}$ is required.

### Solutions in the upper and lower flat surfaces

Noting (8), in the flat area outside the hyperbolic region, where $R'(u)=\pm 1$ for upper and lower planes, respectively, the equations of motion (18) take the form

31 $$\begin{aligned} \begin{aligned}{}&\varphi '' \left( u \right) + \lambda {\frac{\,\varphi ' \left( u \right) }{R }}+\varphi \left( u \right) \left( {k}^{2}-{\frac{ \left( {\lambda } -2\,{m'}\,\sigma \right) ^{2}}{4 R ^{2}}} \right) =0, \end{aligned} \end{aligned}$$

with $\lambda =1$ and $\lambda =-1$ indicate the upper and lower planes, respectively, and $\varphi _{up}$ and $\varphi _d$ are associated with $\sigma =\pm 1$, respectively. Also, the equations (16) and (17) read

32 $$\begin{aligned} \chi _{up} \left( u \right) ={\frac{i}{k} \left( \varphi _d' \left( u \right) +{\frac{ \left( \lambda +2\,{m'} \right) }{2R }}\varphi _d \left( u \right) \right) }, \end{aligned}$$

33 $$\begin{aligned} \chi _d \left( u \right) ={\frac{i}{k} \left( \varphi _{up}' \left( u \right) +{\frac{ \left( \lambda -2\,{m'}\, \right) }{2R }}\varphi _{up} \left( u \right) \right) }. \end{aligned}$$

By solving (31), we obtain the $\varphi (u)$ in terms of the Hankel function of the first and second kind, where for $u \ge u_{p}$ in the upper plane are given by

34 $$\begin{aligned} \begin{aligned} \varphi ^{(p)}(u)=&R^{(p)}_{\sigma } \left( i\sigma -{\textrm{e}^{i\pi \,{m_p'}}} \right) {{ H}^{(1)}_{{m_p'}-\sigma /2}\left( kR \left( u \right) \right) }+I_{\sigma } \left( i\sigma +{\textrm{e}^{-i\pi \, {m_p'}}} \right) {{ H}^{(2)}_{{m_p'}-\sigma /2}\left( kR \left( u \right) \right) }, \end{aligned} \end{aligned}$$

and for $u \le u_{m}$ in the lower plane

35 $$\begin{aligned} \begin{aligned} \varphi ^{(m)}(u)=&R^{(m)}_{\sigma }\left( -i \sigma -i{\textrm{e}^{i\pi \,{m_m'}}} \right) {{ H}^{(1)}_{{m_m'}+\sigma /2} \left( - kR(u) \right) } +T_{\sigma }\left( -i\sigma +{\textrm{e}^{-i\pi \,{m_m'}}} \right) { { H}^{(2)}_{{m_m'}+\sigma /2} \left( -kR(u) \right) }. \end{aligned} \end{aligned}$$

Then, (32) gives the $\chi (u)$ at the upper and lower planes, respectively, by

36 $$\begin{aligned} \begin{aligned} \chi ^{p}(u)=&R^{(p)}_{\sigma } \left( 1- i\sigma {\textrm{e}^{i\pi \,{m_p'}}} \right) { { H}^{(1)}_{{m_p'}-\sigma /2} \left( kR(u) \right) } +I_{\sigma } \left( 1+ i\sigma {\textrm{e}^{-i\pi \,{m_p'}}} \right) {{ H}^{(2)}_{{m_p'}-\sigma /2} \left( kR(u) \right) }, \end{aligned} \end{aligned}$$

37 $$\begin{aligned} \begin{aligned} \chi ^{m}(u)=&R ^{(m)}_{\sigma }\left( 1+i\sigma {\textrm{e}^{i\pi \,m_m'}} \right) {{ H}^{(1)}_{m_m'+\sigma /2}\left( -kR \left( u \right) \right) } +T_{\sigma } \left( 1-i\sigma {\textrm{e}^{-i\pi \,m_m'}} \right) {{ H}^{(2)}_{m_m'+\sigma /2}\left( -kR \left( u \right) \right) }, \end{aligned} \end{aligned}$$

where $\varphi _{up}$ and $\chi _{up}$ ($\varphi _{d}$ and $\chi _{d}$) are associated with $\sigma =1$ ($\sigma =-1$). The Hankel function of the first and second kinds correspond to the waves propagating in the positive and negative directions of *u*, respectively.

### Matching conditions in the scattering

Having found the four components of $\Psi (t,u,v)$ (13), where each of the components is considered to include four terms at the upper and lower flat planes and the curved hyperbolic wormhole throat, the matching conditions at the boundaries that connect these three regions need to be considered. The matching conditions to be employed on the wave functions are the equality of angular momentum ($m'$), energy (*E*), and consequently the momentum *k* due to the relation (20). In addition, the wave functions are required to be smooth continued (i.e., $C^{1}$ continued) at the boundaries connecting three regions of the wormhole.

Our analysis assumes that incoming electrons with energy *E* propagate from the upper graphene layer and interact with the wormhole surface. When the propagating waves in the upper plane, considered to be the incoming waves that scatter with the wormhole, reach the upper boundary at $u_{p}$, the waves will be partially transmitted into the wormhole and partially reflected back. At the lower boundary $u_{m}$, the transmitted waves will be again undergo the partial reflection and transmission. This scenario implies $R^{(m)}_{\sigma }=0$, since it does not include incoming waves at the lower plane. Then, the normalization of $I_{\sigma } \equiv 1$ and $R^{(p)}_{\sigma }\equiv R_{\sigma }$ can be applied, where the outgoing and incoming waves are associated with the Hankel function of the first and second kinds.

Accordingly, by applying $m'_p=m'_m=m'$, as a boundary condition at the upper plane we have

38 $$\begin{aligned} \begin{aligned} \varphi ^{(p)}(u)=&R_{u} \left( i-{\textrm{e}^{i\pi \,{m'}}} \right) {{ H}^{(1)}_{{m'}-\frac{1}{2}}\left( kR \left( u \right) \right) }-R_{d} \left( i+{\textrm{e}^{i\pi \,{m'}}} \right) {{ H}^{(1)}_{{m'}+\frac{1}{2}}\left( kR \left( u \right) \right) }\\&+\frac{1}{\sqrt{2}} \left( i+{\textrm{e}^{-i\pi \, {m'}}} \right) {{ H}^{(2)}_{{m'}-\frac{1}{2}}\left( kR \left( u \right) \right) }+ \frac{1}{\sqrt{2}} \left( -i+{\textrm{e}^{-i\pi \, {m'}}} \right) {{ H}^{(2)}_{{m'}+\frac{1}{2}}\left( kR \left( u \right) \right) }, \end{aligned} \end{aligned}$$

39 $$\begin{aligned} \begin{aligned} \chi ^{p}(u)=&R_{u} \left( 1- i{\textrm{e}^{i\pi \,{m'}}} \right) { { H}^{(1)}_{{m'}-\frac{1}{2}} \left( kR(u) \right) } +R_{d} \left( 1+ i{\textrm{e}^{i\pi \,{m'}}} \right) { { H}^{(1)}_{{m'}+\frac{1}{2}} \left( kR(u) \right) }\\ {}&+\frac{1}{\sqrt{2}} \left( 1+ i{\textrm{e}^{-i\pi \,{m'}}} \right) {{ H}^{(2)}_{{m'}-\frac{1}{2}} \left( kR(u) \right) } +\frac{1}{\sqrt{2}} \left( 1- i{\textrm{e}^{-i\pi \,{m'}}} \right) {{ H}^{(2)}_{{m'}+\frac{1}{2}} \left( kR(u) \right) }, \end{aligned} \end{aligned}$$

while at the lower plane

40 $$\begin{aligned} \begin{aligned} \varphi ^{(m)}(u)=&T_{u}\left( -i+{\textrm{e}^{-i\pi \,{m'}}} \right) { { H}^{(2)}_{{m'}+\frac{1}{2}} \left( -kR(u) \right) } +T_{d}\left( i+{\textrm{e}^{-i\pi \,{m'}}} \right) { { H}^{(2)}_{{m'}-\frac{1}{2}} \left( -kR(u) \right) }, \end{aligned} \end{aligned}$$

41 $$\begin{aligned} \begin{aligned} \chi ^{m}(u)=&T_{u} \left( 1-i {\textrm{e}^{-i\pi \,m'}} \right) {{ H}^{(2)}_{m'+\frac{1}{2}}\left( -kR \left( u \right) \right) }+T_{d} \left( 1+i {\textrm{e}^{-i\pi \,m'}} \right) {{ H}^{(2)}_{m'-\frac{1}{2}}\left( -kR \left( u \right) \right) }. \end{aligned} \end{aligned}$$

where $R_{u}$ and $R_d$ are the reflection coefficients and $T_u$ and $T_d$ are transmission coefficients for up and down spins, respectively.

### T-matrix

Aimed at investigating the spin-dependent transmission probabilities and GMR of a single layer of wormhole graphene under the external magnetic field with constant flux, the *T*-matrix method^35^ can be utilized here to obtain the transmission and reflection probabilities.

The *T*-Matrix method is a mathematical technique used to analyze the propagation of waves through layered structures. It is commonly employed in optics, electromagnetics, and solid-state physics to study the transmission and reflection properties of multilayer systems. Once the overall transfer matrix is obtained, it can be used to calculate various properties of interest, such as the transmission and reflection coefficients, GMR, the phase shift, and the intensity distribution within the structure. By manipulating the transfer matrix or modifying the properties of individual layers, researchers can analyze and optimize the performance of multilayer systems, such as optical coatings, photonic devices, or thin film structures.

Here, considering the wave functions of the upper plane, given by (38) and (39), at the upper boundary $u=u_p$, where $R(u)=R_p$ and the $R_p$ is given by (9), we define the following matrix

42 $$\begin{aligned} N={\textrm{e}^{i \left( m'+{\frac{\phi }{\phi _0}} \right) v}}\left[ \begin{array}{cccc} H^{(2)}_{-}&{}i{\textrm{e}^{-i m' \,\pi }}{ H^{(2)}_{-}}&{}H^{(1)}_{-}&{}-i{\textrm{e}^{i m' \,\pi }}H^{(1)}_{-}\\ H^{(2)}_{+}&{}-i{\textrm{e}^{-i m' \,\pi }}H^{(2)}_{+}&{}H^{(1)}_{+}&{}i{\textrm{e}^{im'\,\pi }}H^{(1)}_{+}\\ iH^{(2)}_{-}&{}{\textrm{e}^{-i m' \, \pi }}H^{(2)}_{-}&{}iH^{(1)}_{-}&{}-{\textrm{e}^{i m' \,\pi }}H^{(1)}_{-} \\ -iH^{(2)}_{+}&{}{\textrm{e}^{-i m' \,\pi }}H^{(2)}_{+}&{}-i{ H^{(1)}_{+}}&{}-{\textrm{e}^{i m' \,\pi }}H^{(1)}_{+}\end{array} \right] , \end{aligned}$$

in which $H^{(i)}_{\pm }={{ H}^{(i)}_{m'\pm 1/2}\left( { kR_p}\right) }$ with $i=1,2$. Also, for the lower plane, at the boundary $u=u_m$, where $R(u)=R_m$ and the $R_m$ is given by (9), we define based on (40) and (41)

43 $$\begin{aligned} M={\textrm{e}^{i \left( m'+{\frac{\phi }{\phi _0}} \right) v}}\left[ \begin{array}{cccc} H^{(2)}_{+}&{}-i{\textrm{e}^{-i m' \,\pi }}{H^{(2)}_{+}}&{}0&{}0\\ H^{(2)}_{-}&{}i{\textrm{e}^{-i m' \,\pi }}{{H^{(2)}_{-}}}&{}0&{}0\\ -iH^{(2)}_{+}&{}{\textrm{e}^{-i m' \,\pi }}{{H^{(2)}_{+}}}&{}0&{}0\\ iH^{(2)}_{-}&{}{\textrm{e}^{-i m' \,\pi }}{{H^{(2)}_{-}}}&{}0&{}0\end{array} \right] , \end{aligned}$$

in which $H^{(i)}_{\pm }={{ H}^{(i)}_{m'\pm 1/2}\left( -{ kR_m}\right) }$ with $i=1,2$.

Furthermore, for the hyperbolic wormhole, considering the wave functions (22), we consider the terms accompanied with $A_{\kappa _{1},\kappa _{2},\sigma }$ and $B_{\kappa _{1},\kappa _{2},\sigma }$ in (22) as $\varphi ^1(\kappa _{1},\kappa _{2})$ and $\varphi ^2(\kappa _{1},\kappa _{2})$, respectively. Then, we define $h_1= \varphi ^1_{up}(+,-)$, $h_2= \varphi ^1_{up}(-,+)$, $h_3= \varphi ^2_{up}(+,-)$, $h_4= \varphi ^2_{up}(-,+)$, $g_1= \varphi ^1_{d}(+,-)$, $g_2= \varphi ^1_{d}(-,+)$, $g_3= \varphi ^2_{d}(+,-)$, $g_4= \varphi ^2_{d}(-,+)$. Also considering the wave functions (25), we consider the terms accompanied with $A_{\kappa _{1},\kappa _{2},\sigma }$ and $B_{\kappa _{1},\kappa _{2},\sigma }$ in (22) as $\chi ^1(\kappa _{1},\kappa _{2})$ and $\chi ^2(\kappa _{1},\kappa _{2})$, respectively. Then, we set $k_1= \chi ^1_{up}(+,-)$, $k_2= \chi ^1_{up}(-,+)$, $k_3= \chi ^2_{up}(+,-)$, $k_4= \chi ^2_{up}(-,+)$, $f_1= \chi ^1_{d}(+,-)$, $f_2= \chi ^1_{d}(-,+)$, $f_3= \chi ^2_{d}(+,-)$, $f_4= \chi ^2_{d}(-,+)$. Accordingly, the following matrix can be considered for the hyperbolic wormhole

44 $$\begin{aligned} K={\textrm{e}^{i \left( m'+{\frac{\phi }{\phi _0}} \right) v}}\left[ \begin{array}{cccc} { k_1} \left( X \right) &{}{ k_2} \left( X \right) &{}{ k_3} \left( X \right) &{}{ k_4} \left( X \right) \\ { f_1} \left( X \right) &{}{ f_2} \left( X \right) &{}{ f_3} \left( X \right) &{}{ f_4} \left( X \right) \\ { h_1} \left( X \right) &{}{ h_2} \left( X \right) &{}{ h_3} \left( X \right) &{}{ h_4} \left( X \right) \\ { g_1} \left( X \right) &{}{ g_2} \left( X \right) &{}{ g_3} \left( X \right) &{}{ g_4} \left( X \right) \end{array} \right] . \end{aligned}$$

The matrix will be evaluated at the upper and lower boundaries, where at $u=u_p$ and $u=u_m$, given by (9), we have $X_p=\frac{r}{a}$ and $X_m=-\frac{r}{a}$, respectively.

Then, the boundary conditions requires

45 $$\begin{aligned} N.\left[ \begin{array}{c} \frac{1}{\sqrt{2}}\\ \frac{1}{\sqrt{2}}\\ R_{u}\\ R_d\end{array} \right] =K\mid _{X=Xp}. \left[ \begin{array}{c} A_1\\ A_2\\ B_1\\ B_2\end{array} \right] , \quad \quad \textrm{and}\quad \quad K\mid _{X=Xm}. \left[ \begin{array}{c} A_1\\ A_2\\ B_1\\ B_2\end{array} \right] = M. \left[ \begin{array}{c} {T_u}\\ {T_d} \\ 0\\ 0\end{array} \right] , \end{aligned}$$

Also, $A_1=A_{+,-,+}$, $A_2=A_{-,+,+}$, $B_1=B_{+,-,+}$, and $B_2=B_{-,+,+}$. According to (45), we can also have

46 $$\begin{aligned} \left( N^{-1}.K\mid _{X=Xp}\right) .\left( K^{-1}. M\mid _{X=Xm}\right) .\left[ \begin{array}{c} {T_u}\\ {T_d} \\ 0\\ 0\end{array} \right] = \left[ \begin{array}{c} \frac{\sqrt{2}}{2}\\ \frac{\sqrt{2}}{2}\\ R_{u}\\ R_d\end{array} \right] . \end{aligned}$$

The provided equations can be used to obtain the $T_u$, $T_d$, $R_u$ and $R_d$. Then, either of equations in (45) can be used to identify the $A_1$, $A_2$, $A_3$, and $A_4$ coefficients.

Using the $T_d$ and $T_u$ obtained via *T*-matrix method, we can determine the GMR as

47 $$\begin{aligned} GMR= (T_u-T_d)/(T_u+T_d) \end{aligned}$$

The study of GMR in quantum structures has garnered significant attention due to its potential applications in spintronics and magnetic memory devices. GMR refers to the large change in electrical resistance observed when a magnetic field is applied to a layered structure containing magnetic and non-magnetic materials. GMR effects in nano systems can be used to create spin filters, spin valves, and other spintronic devices that utilize the spin of electrons for information storage and processing.

## Results and discussion

In this section, we present the numerical results obtained for the described graphene wormhole structure in the following figures.

**Figure 2:**
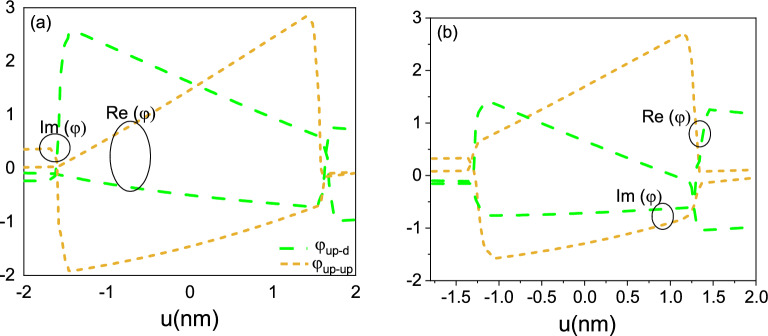
Real and imaginary parts of wave function $\varphi _{up}$ as the function of *u* for (**a**) radius $a = 8nm$, (**b**) $a = 10nm$.

Studying wave functions in nano structures is crucial for understanding the energy levels, size and shape effects, electron behavior, device design, and fundamental quantum mechanics associated with these nanostructures. The knowledge gained from such studies enables the development of novel technologies and applications based on quantum structures. Researchers often employ theoretical models to describe the behavior of electrons in quantum structures. These models, such as the effective mass approximation or the density functional theory, provide mathematical frameworks to calculate and predict wave functions. In cases where exact analytical solutions are not feasible, numerical simulations are employed. Experimental techniques like scanning probe microscopy can provide direct information about the wave functions in quantum structures. By combining theoretical calculations with experimental techniques, researchers can analyze and determine the wave functions in quantum structures. These approaches provide valuable insights into the electronic properties, behavior of electrons, and energy levels in these nano-scale systems. In Fig. 2, we show the real and imaginary parts of $\varphi _{up-up}$ with and without spin-flip $\varphi _{up-d}$ for $m=1$ and (a) $a=8nm$ and (b) $a=10nm$. As mentioned, $\varphi _{up}>$ and $\chi _{d}>$ are coupled to each other, so describing the behavior of a single component of such a four-component wave function is very complicated.

**Figure 3:**
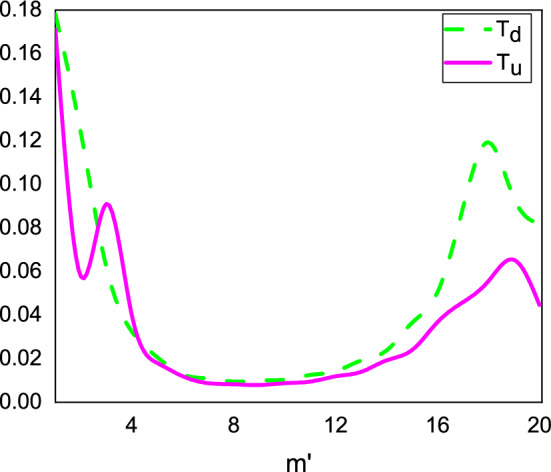
Transmission coefficients $T_u$ and $T_d$ as the function of the effective angular quantum number $m'$ for $r=3$ nm and $a=5$ nm.

Figure 3 displays the dependence of the transmission coefficients $T_u$ and $T_d$ on the effective angular quantum number $m'$. The coupling between spin-up and spin-down wave functions is due to the wormhole structure. This coupling can also arise due to the presence of magnetic fields or other interactions that can affect the spin of the particles. The coupling between spin-up and spin-down wave functions leads to the spin-up and spin-down states being related to each other and cannot be treated as independent. The coupling between spin-up and spin-down wave functions is a fundamental aspect of quantum mechanics that has important consequences for the behavior of many physical systems. One example of a spintronics device that utilizes the coupling between spin-up and spin-down wave functions is the magnetic tunnel junction (MTJ). The coupling between spin-up and spin-down wave functions means that the spin-up and spin-down states are related to each other and cannot be treated as independent in some systems. In other words, the behavior of one spin state can affect the behavior of the other spin state. There are several practical applications of the coupling between spin-up and spin-down wave functions, especially in the field of spintronics such as Magnetic memory devices, Spin filters, and Spin qubits. Our findings indicate that as the value of $m'$ increases, the transmission probabilities initially decrease until reaching a minimum, after which they increase again for high values of $m'$. Moreover, we observed that the probability of spin-flip is almost larger than that of non-spin-flip, indicating that electrons lose their spins during transmission. This feature is particularly important for magnetic information storage and fabrication. The observed $m'$-dependence of the transmission probabilities can be attributed to the interference between the incoming and reflected waves at the upper and lower Hilbert horizons, which leads to resonant transmission and reflection for certain values of $m'$. The spin-flip effect, on the other hand, arises from the presence of the magnetic field, which causes the spin orientation of the electrons to process as they propagate through the material. Overall, our results suggest that wormhole graphene under external magnetic fields can exhibit interesting spin-dependent transport properties that may have potential applications in spintronics and magnetic information storage.

**Figure 4:**
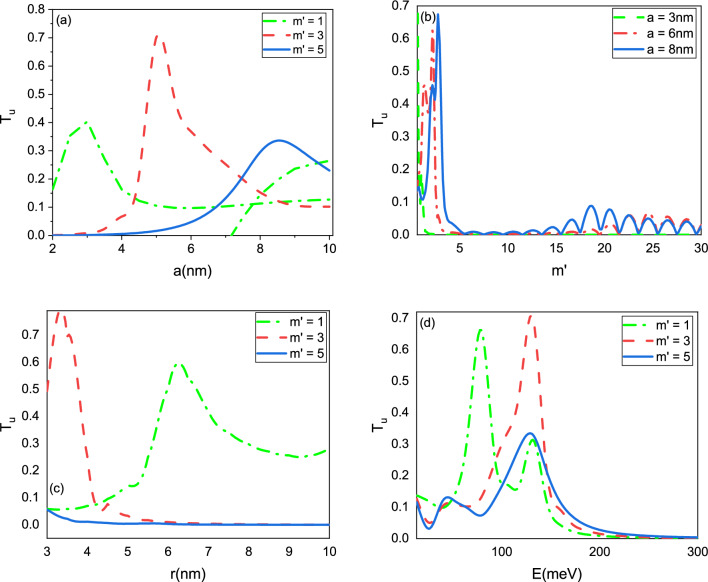
Transmission probability $T_u$ as the function of the (**a**) radius *a*, (**b**) angular quantum number $m'$, (**c**) radius *r*, and (**d**) energy *E*.

Figure 4 illustrates the dependence of the transmission probability $T_u$ on the angular quantum number $m'$, energy *E*, and radii *a* and *r*. Our results demonstrate that these parameters can strongly influence the transport properties of the wormhole graphene system. As expected, we found that $T_u$ decreases as the value of $m'$ increases, reflecting the interference between the incoming and reflected waves at the Hilbert horizons. We also observed that the transmission probability increases as the radius *a* of the wormhole increases, due to the increased cross-sectional area available for electron transmission. Conversely, the transmission probability decreases as the length of the wormhole increases, which occurs when the radius *r* increases. This effect can be attributed to the increased amount of scattering and reflection that occurs as the electrons propagate over a longer distance. The higher energies lead to a decrease in the radius *a*. This decrease in radius can in turn lead to a decrease in the transmission probabilities of particles. Together, our results suggest that the transport properties of wormhole graphene can be effectively controlled by tuning the angular quantum number, energy, and geometric parameters of the system. These findings may have potential applications in the design of novel electronic and spintronic devices.

**Figure 5:**
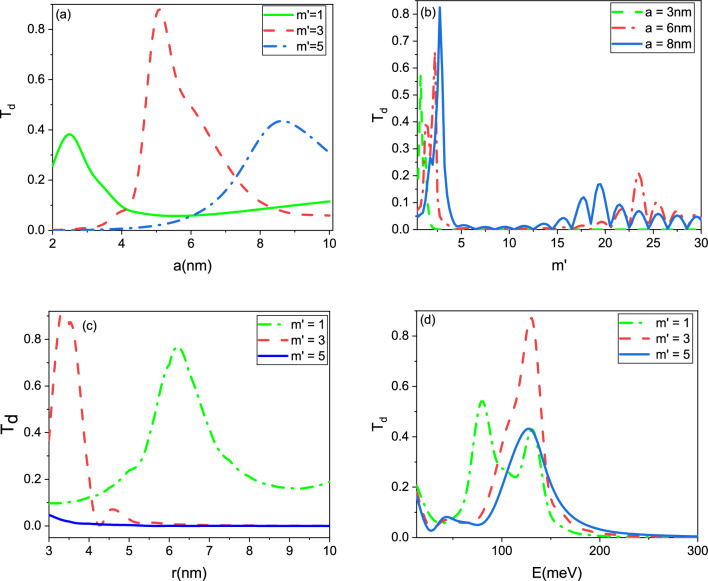
Transmission probability $T_d$ as the function of the (**a**) radius *a*, (**b**) angular quantum number $m'$, (**c**) radius *r*, and (**d**) energy *E*.

In addition to the results obtained for $T_u$, we also investigated the behavior of the transmission coefficient $T_d$ as a function of the angular quantum number $m'$, energy *E*, and radii *a* and *r*, as depicted in Fig. 5. Interestingly, we observed a different behavior for $T_d$ compared to $T_u$, suggesting that the spin-dependent transport properties of the wormhole graphene system are highly sensitive to the specific details of the system. Our findings suggest that by applying an external magnetic field to the wormhole graphene system, it is possible to further control the transmission probabilities and spin-dependent transport properties. Additionally, modifying the geometry of the wormhole, such as introducing additional constrictions or widenings, can lead to resonant transmission or reflection for specific values of energy or angular momentum. Overall, these results highlight the rich and complex behavior of wormhole graphene under external magnetic fields, and suggest that this material may have potential applications in spintronics and electronic devices. The ability to tune and control the transport properties of the system through external and internal parameters may enable the development of novel devices with enhanced functionality and performance.

While the authors showed that $T(m')$ and $R(m')$ exhibit oscillating behavior for $m'$^26^, we found that the transmission coefficients exhibit a different behavior. The reason may lie in the fact that we have not used the asymptotic forms of the wave functions at the boundaries, and not only the $\textrm{e}^{im v}$ plays the $m'$-dependence role, but also the Jacobi polynomials contributed significantly in our analysis. The spin-dependent transmission coefficient plots provide valuable tool for analyzing and designing spin-dependent transport systems. These plots provide information on how the transmission of particles with different spin orientations changes as a function of the parameters of the system. This information is important for understanding the behavior of spin-dependent transport in various materials and devices, and for designing new materials and devices with specific spin-dependent properties. In a system where the spin-dependent transmission coefficient is present, the transmission of particles with different spin orientations can be influenced differently. By adjusting the parameters that affect the spin-dependent transmission coefficient, we can manipulate the spin transport properties of a system and thus design devices with desired functionalities. The spin-dependent transport properties of a graphene wormhole can be controlled by adjusting the size and shape of the hole, the spin-orbit coupling strength, and the magnetic field applied to the system.

**Figure 6:**
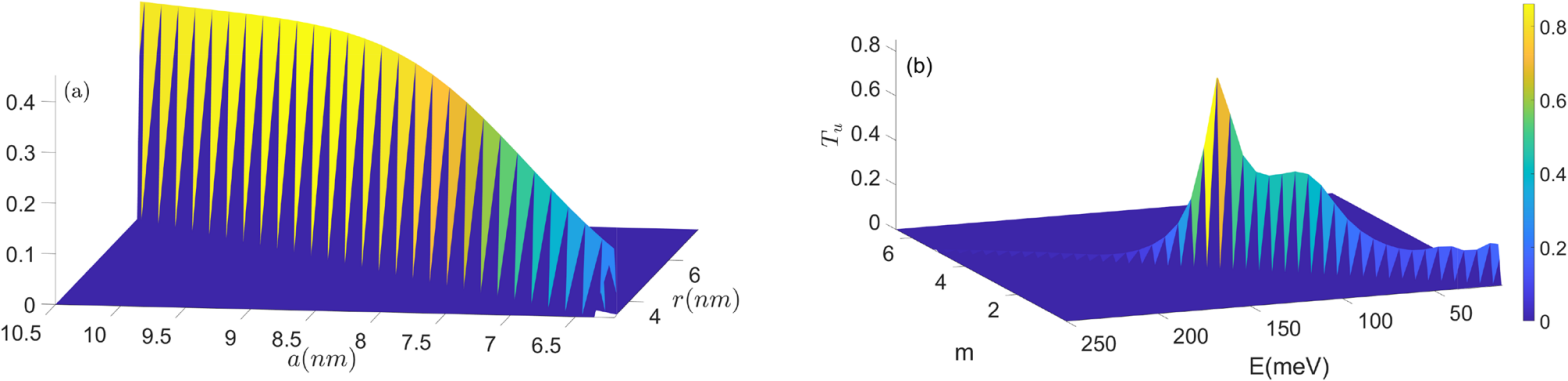
Transmission probability $T_u$ as the function of the (**a**) radii *a* and *r*, (**b**) angular quantum number $m'$ and energy *E*.

**Figure 7:**
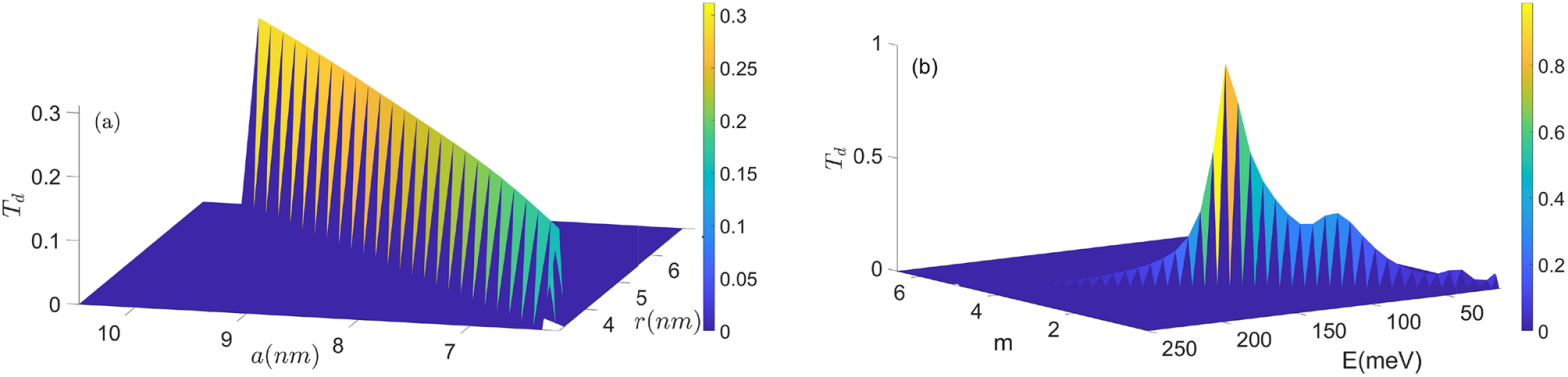
Transmission probability $T_d$ as the function of the (**a**) radii *a* and *r*, (**b**) angular momentum number $m'$ and energy *E*.

In Figs. 6 and 7, $T_u$ and $T_d$ are plotted as a function of radii *a* and *r* in panel (a), and as a function of *m* and *E* in panel (b). As expected, $T_d$ is higher than $T_u$ with increasing parameter $m'$. While $T_d$ is lesser than $T_u$ with increasing radii *a* and *r*. These parameters can effectively control the spin-dependent transmission coefficient.

**Figure 8:**
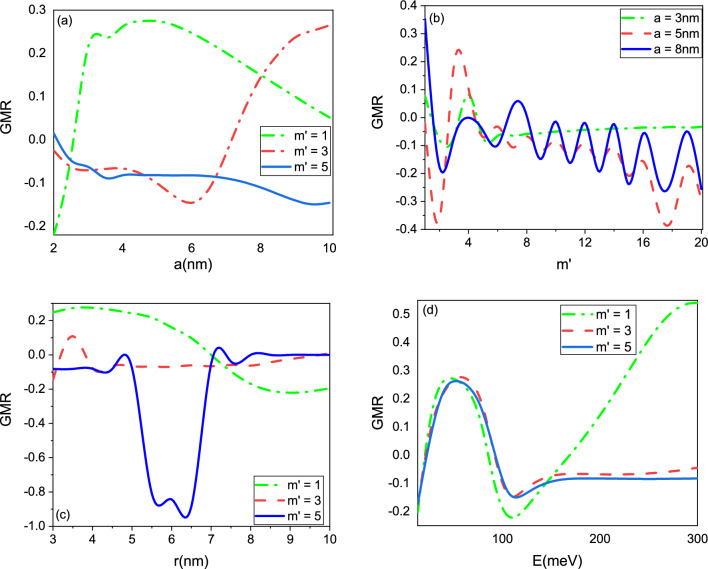
GMR as the function of the (**a**) radius *a*, (**b**) angular quantum number $m'$, (**c**) radius *r*, and (**d**) energy *E*.

In the following, we investigated the effects of wormhole size, incident energy, and magnetic field on GMR. In general, the GMR effect arises from the spin-dependent scattering of electrons at interfaces between magnetic and non-magnetic materials. The size of the wormhole can affect the electronic properties of the material and the spin-dependent scattering of electrons, which can in turn affect the magnitude of the GMR effect. The incident energy of the electrons can also affect the GMR effect, as higher energy *E*lectrons can penetrate deeper into the material and interact differently with the magnetic and non-magnetic regions. Finally, the magnetic field can directly influence the GMR effect by altering the relative orientation of the magnetic and non-magnetic regions and thus affecting the spin-dependent scattering of electrons. Overall, the effects of wormhole size, incident energy, and magnetic field on GMR are complex and depend on the specific system under study. Further research is needed to fully understand the interplay between these parameters and their effects on GMR in various spintronics devices. Figure 8 shows that the size of the wormhole, incident energy, and magnetic field can all simultaneously affect the GMR. The size of the wormhole can affect the electronic properties of the material and thus the magnitude of the GMR effect. We observed that as the radius of the wormhole increases, the GMR exhibits oscillatory behavior (Fig. 7a) and as *r* increases, GMR decreases (Fig. 7c). Previous studies have shown that there are several other factors that can affect the oscillatory behavior of GMR. In addition to the size of the wormhole, other factors that can influence the oscillations in GMR include the strength and direction of the magnetic field, the spin polarization of the injected current, and the geometry of the device^36,37^. The energy of the electrons can affect the GMR effect by altering the penetration depth of the electrons into the magnetic and non-magnetic regions of the material. Higher energy *E*lectrons can penetrate deeper into the material, and thus the GMR effect can be affected by the energy of the electrons. The strength and direction of the magnetic field can directly influence the magnitude of the GMR effect. When the magnetic field is applied perpendicular to the plane of the material, the GMR effect is maximized. The GMR effect decreases as the angle between the magnetic field and the plane of the material increases. As the energy increases from zero to 100, GMR exhibits a similar behavior for $m'$ values of 1, 3, and 5 (Fig. 7d). For higher magnetic fields $(m'=2, 3)$, GMR remains nearly constant, while for weaker magnetic fields, GMR increases. As the energy of the electrons in the device increases, the spin-dependent scattering of the electrons by the magnetic fields becomes more efficient, which results in an increase in GMR. This behavior is due to the fact that the scattering of the carriers is strongly dependent on the relative orientation of the magnetic field in the graphene layers.

The magnetic field can affect the GMR effect in several ways. First, the magnetic field can directly influence the orientation of the magnetic moments in the magnetic and non-magnetic regions of the material. When the magnetic field is applied perpendicular to the plane of the material, the magnetic moments in the magnetic and non-magnetic regions become aligned, resulting in an increase in the GMR effect. Conversely, when the magnetic field is applied parallel to the plane of the material, the magnetic moments become anti-aligned, resulting in a decrease in the GMR effect. Second, the magnetic field can alter the spin-dependent scattering of electrons passing through the material. In a typical GMR device, a current of spin-polarized electrons is injected into the magnetic layer, where it interacts with the magnetic moments. The scattered electrons then pass through a non-magnetic layer, where their spin polarization is detected. The magnetic field can affect the spin-dependent scattering of electrons by altering the relative orientation of the magnetic moments in the magnetic layer and thus affecting the probability of electron scattering. Finally, the strength of the magnetic field can also affect the magnitude of the GMR effect. In general, a stronger magnetic field can result in a larger GMR effect by increasing the degree of magnetic moment alignment in the magnetic and non-magnetic regions of the material.

## Conclusion

We have studied the spin-dependent quantum transport of massless Dirac fermion on $(2+1)$-dimensional curved spacetime in the presence of constant axial magnetic flux is explored. The geometric and gauge setup provided in^26^ has been used, where the geometry of spatial part of spacetime is considered to be a wormhole created by connection of a hyperbolic bridge between two upper and lower plane, while a constant magnetic flux is applied. In order to employ the *T*-matrix method to study the transmission probability, we found four-component Dirac spinor as the solution of Dirac equation on curved spacetime. The class of solutions associated with real energy and momentum are then chosen to be utilized. The coupling between spin-up and spin-down wave functions is due to the wormhole structure. Also, the magnetic field enhances this coupling and thus causes the spin orientation of the electrons to precess as they propagate through the material. Our numerical results indicate that the magnetic field, incident energy and wormhole dimension can effectively control the spin-dependent transport properties.
